# Apoptosis in the aging liver

**DOI:** 10.18632/oncotarget.21123

**Published:** 2017-09-21

**Authors:** Hua-Hua Zhong, Shao-Jie Hu, Bo Yu, Sha-Sha Jiang, Jin Zhang, Dan Luo, Mei-Wen Yang, Wan-Ying Su, Ya-Lan Shao, Hao-Lin Deng, Fen-Fang Hong, Shu-Long Yang

**Affiliations:** ^1^ Department of Physiology, College of Medicine, Nanchang University, Nanchang 330006, China; ^2^ Department of Experimental Teaching Center, Nanchang University, Nanchang 330031, China

**Keywords:** apoptosis, aging, liver, oxidative stress, genomic instability

## Abstract

Various changes in the liver during aging can reduce hepatic function and promote liver injury. Aging is associated with high morbidity and a poor prognosis in patients with various liver diseases, including nonalcoholic fatty liver disease, hepatitis C and liver cancer, as well as with surgeries such as partial hepatectomy and liver transplantation. In addition, apoptosis increases with liver aging. Because apoptosis is involved in regeneration, fibrosis and cancer prevention during liver aging, and restoration of the appropriate level of apoptosis can alleviate the adverse effects of liver aging, it is important to understand the mechanisms underlying this process. Herein, we elaborate on the causes of apoptosis during liver aging, with a focus on oxidative stress, genomic instability, lipotoxicity, endoplasmic reticulum stress, dysregulation of nutrient sensing, and liver stem/progenitor cell activity.

## INTRODUCTION

As a natural aspect of life, aging is the biological process in which a person gradually loses the ability to maintain homeostasis, physiological function, proliferation and immune responses. During liver aging, a series of physiological and pathological alterations occur, such as reduced blood perfusion, diminished metabolism and increased susceptibility to liver fibrosis and hepatocarcinoma. Consequently, myriad stress stimulators induce cellular deterioration. As many aging-related stressors are potentially oncogenic, cancer is likely to occur if this deterioration persists long enough. Thus, in liver cells, apoptosis or senescence may be tumor-protective mechanisms to maintain homeostasis during aging.

Apoptosis (or programmed cell death) is a highly regulated process in which a cell degrades DNA and proteins and subsequently breaks into fragments known as apoptotic bodies, which are engulfed by nearby phagocytic cells and quickly removed without eliciting any inflammatory response. During liver aging, excessive apoptosis has been identified in non-alcoholic and alcoholic liver disease, acute and chronic viral hepatitis, and cholestatic liver disease. Sustained apoptosis has also been linked with the development of hepatic fibrosis. On the other hand, insufficient apoptosis has been associated with the development and progression of tumors of the liver and the biliary tree [1].

It is likely that cells can only choose one fate between apoptosis and senescence; once cellular senescence is established, cells become resistant to apoptosis, and vice versa [2]. In contrast to apoptotic cells, senescent cells are stably viable and can influence neighboring cells by secreting a suite of cytokines, chemokines and matrix-remodeling enzymes, collectively known as the senescence-associated secretory phenotype. Although this phenotype has been associated with tissue and organ deterioration and possibly even tumor growth, senescent cells are also present early in life and are largely beneficial for homeostasis and development [3]. Thus, it is worth examining the relationship between apoptosis and senescence.

Whether and how apoptosis increases or decreases during liver aging remains a matter of debate. On the one hand, ineffective clearance of apoptotic bodies by neighboring phagocytes can lead to severe liver damage in viral hepatitis, and excessive hepatocyte apoptosis can aggravate hepatic fibrosis and cirrhosis. On the other hand, deficient apoptosis contributes to the development of liver and biliary cancer [1, 4]. Salminen, Ojala and Kaarniranta suggested that aging suppresses apoptosis due to functional deficiencies in the p53 network, increased activity in the nuclear factor kappa B (NF-κB)/inhibitor of apoptosis protein (IAP)/c-Jun N-terminal kinase (JNK) axis, and changes in molecular chaperones, microRNAs and epigenetic regulation. Moreover, this suppression of apoptosis enhances the aging process [5]. However, Childs *et al.* indicated that the aging-related inclination to apoptosis is cell-type specific; for instance, senescent endothelial cells are susceptible to apoptosis, whereas senescent fibroblasts and keratinocytes are likely to escape apoptotic death [3].

Promisingly, interventional apoptosis modulation is being exploited to combat aging. Chang *et al.* reported that ABT263 (a specific inhibitor of the anti-apoptotic proteins B-cell lymphoma 2 [BCL-2] and BCL-xL) selectively killed senescent cells in aged mice and rejuvenated hematopoietic stem cells [6]. A recent study also indicated that targeted apoptosis of senescent cells in aged mice could restore tissue homeostasis [7]. In addition, apoptosis is recognized as an anti-cancer mechanism in aging, while its deregulation is a hallmark of cancer, implying that apoptosis restoration could be a novel method of preventing cancer [8]. Thus, it is essential to understand the mechanisms of apoptosis in liver aging, especially for the sake of future apoptosis therapy.

To date, many studies on apoptosis in liver aging have been conducted, but the mechanisms underlying this process have yet to be summarized. In order to fill this gap, we have reviewed the recent advances in this field as follows.

### Oxidative stress and apoptosis in liver aging

Oxidative stress is theorized to reflect an imbalance between the synthesis of reactive oxygen species (ROS) and the ability of an organism to detoxify the reactive intermediates or repair the resulting damage. Over the last 20 years, this theory has gradually shifted from the notion that ROS damage normal cellular structures and alter their functions in a way that accelerates aging, to the hypothesis of hormesis – that is, that ROS have two faces, and their ultimate effects depend on the exposure time and dose. Since the mitochondrion is the center of oxidative phosphorylation and ATP production, ROS are primarily generated when mitochondrial dysfunction occurs.

In the traditional view, aging-related mitochondrial dysfunction perturbs the well-functioning electron transport chain, such that oxygen molecules are not fully reduced and thus become ROS, causing DNA damage, protein crosslinking and lipid peroxidation. These changes further damage the mitochondrion, thus forming a vicious cycle. The present view, on the other hand, also considers ROS as signaling molecules that regulate biological and physiological processes, such as proliferation and tissue maintenance. Moderate ROS levels can dampen tissue degeneration, thereby promoting healthy aging, whereas excessive ROS levels can break the balance in the oxidant-antioxidant system, causing oxidative stress and accelerated aging [9].

**Figure 1:**
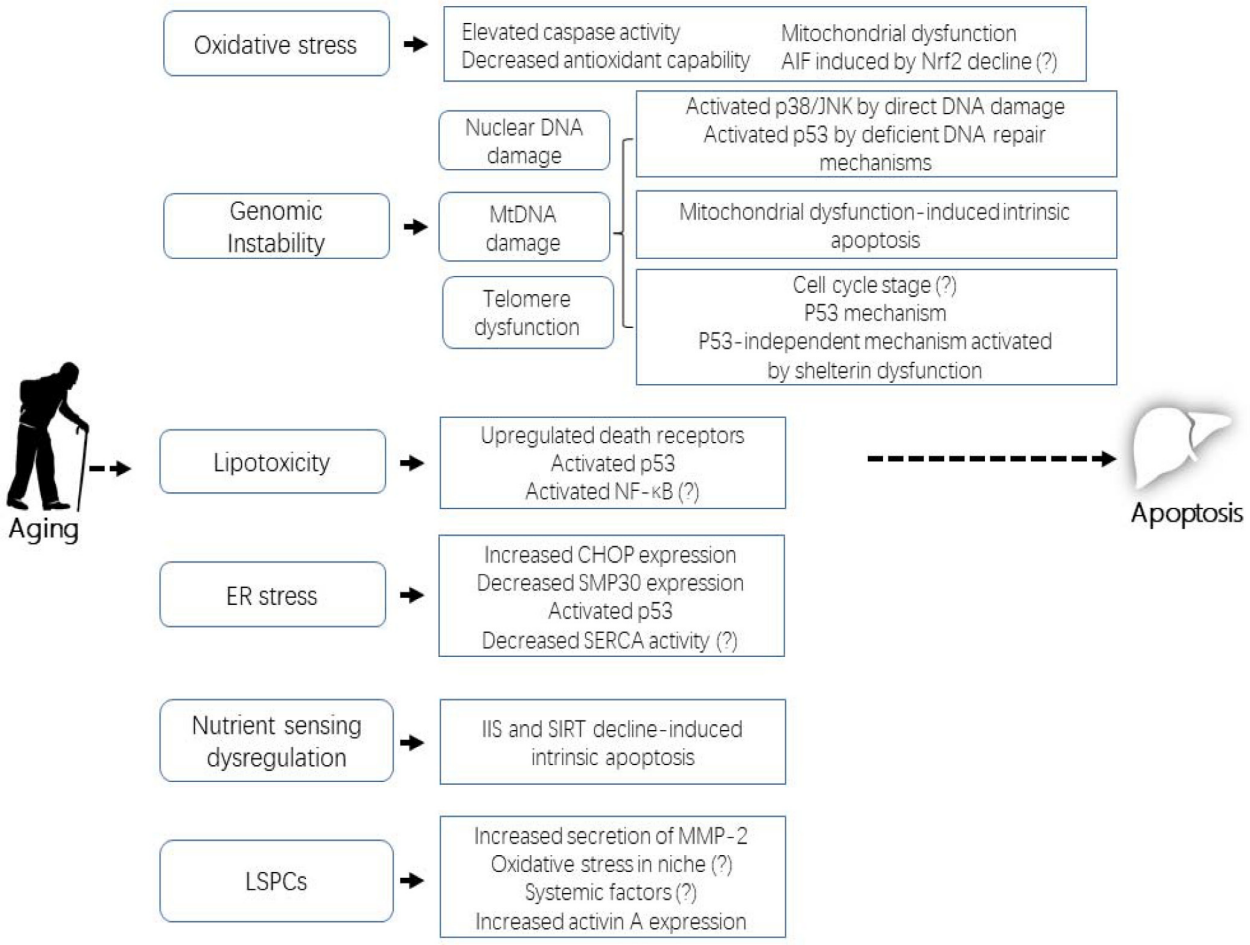
Mechanisms of apoptosis in liver aging

Damage inflicted on liver cells by accumulated ROS increases with aging [10]. For instance, more oxidative damage has been found in the livers of normally aging rhesus monkeys and mice than in those of their younger counterparts [11, 12]. The mechanisms of oxidative stress-induced apoptosis in liver aging involve elevated caspase activity, a reduced antioxidant capability, mitochondrial dysfunction, and apoptosis-inducing factor (AIF) activity induced by declining nuclear factor E2-related factor-2 (NRF2) levels (Figure 1).

Caspases consist of initiators and executioners in the classical apoptotic pathway, and are constitutively expressed as monomeric inactive zymogens in the cytosol, but can be activated by ROS via proteolysis at internal sites [13]. Zhang *et al.* found that the senescent liver cells of normally aging Fisher 344 rats were more sensitive to oxidative stress and underwent more apoptosis than those of young rats. In these liver cells, the activity of mitochondrial (i.e., intrinsic) pathway-related caspases-2, –3, –6, –7 and –9 was increased, but the activation of caspase-8, a key enzyme for extrinsic apoptosis, was not detected, suggesting that extrinsic apoptosis may not be a major contributor to normal liver aging [14].

During aging, declines in numerous endogenous antioxidant systems [15] and abnormalities in mitochondrial function not only contribute to aging-related oxidative stress and apoptosis, but also influence each other: (1) a reduced antioxidant capability can cause ROS accumulation, leading to the collapse of the mitochondrial membrane potential, the mitochondrial translocation of BCL-2-associated X (BAX) and BCL-2-associated agonist of cell death (BAD), the formation of the mitochondrial permeability transition pore (mtPTP) and the release of pro-apoptotic factors like cytochrome c to initiate intrinsic apoptosis; (2) significant mitochondrial loss of cytochrome c will disrupt the electron transport chain, leading to further ROS accumulation; (3) ROS can also activate the JNK pathway via apoptosis signal-regulating kinase 1 (a redox-sensitive mitogen-activated protein kinase kinase kinase) to induce extrinsic or intrinsic apoptotic signaling; (4) a reduced antioxidant capability can even cause ROS-independent apoptosis, for instance, due to glutathione efflux [16]. Both in mice deficient in the antioxidant protein thioredoxin 2 [17] and in mice deficient in the antioxidant enzyme manganese superoxide dismutase [18], the hepatic levels of oxidative stress products and apoptosis were significantly greater than in control mice. The livers of thioredoxin 2 (+/–) mice exhibited increased oxidative damage and reduced mitochondrial function, both of which contributed to apoptosis. As for manganese superoxide dismutase, partial or complete deficiency of this enzyme in mice caused age-related increases in mitochondrial oxidative stress in the liver, thus increasing the sensitization of the mtPTP, stimulating the release of pro-apoptotic factors from mitochondria into the cytoplasm and prematurely inducing apoptosis. On the contrary, in a D-galactose-induced aging mouse model, treatment with antioxidant medicines like silybum marianum oil [19] or polydatin [20] reduced the hepatic levels of oxidative stress products, apoptosis and caspase-3, but increased the hepatic levels of the anti-apoptotic protein BCL-2.

NRF2 signaling, which upregulates the basal and inducible expression of many antioxidant and proteasomal enzymes that attenuate oxidative stress, also declines with liver aging [21]. Ariza *et al.* found that mitochondrial function was abnormal, mitochondrial permeabilization was reduced and basal apoptosis was increased in liver tissue from *Nrf2* knockout mice, by a caspase-independent and AIF-dependent pathway [22], implying that AIF might stimulate apoptosis during liver aging. In this case, AIF moved from mitochondria to the nucleus and triggered apoptosis via chromatin condensation and DNA fragmentation without activating caspases.

The scheme describes the six basic aspects introduced in this review: oxidative stress, genomic instability, lipotoxicity, endoplasmic reticulum (ER) stress, dysregulation of nutrient sensing, and liver stem/progenitor cell (LSPC) activity. Note that a question mark following a specific regulatory mechanism indicates that, based on the existing evidence, we can only postulate instead of assert that it contributes to apoptosis in liver aging.

### Genomic instability and apoptosis in liver aging

Genomic instability has long been recognized as a causal factor in aging. The liver is characterized by a high incidence of carcinogenesis with aging. During this process, alterations in vascular structure, declines in normal functioning, and age-related liver disease jointly cause genomic stress. Genomic stress, in turn, increases the susceptibility of hepatic DNA to a variety of internal and exogenous attacks, such as hydrolysis, oxidation, spontaneous alkylation, and ionizing and ultraviolet radiation [23]. As a result, many kinds of DNA lesions occur, all of which impede the normal function of DNA and require correction by processes such as base excision repair, nucleotide excision repair and double-strand break repair. Sensor systems, such as the main DNA damage recognition factor (the MRN [Mre11-Rad50-Nbs1] complex) and the phosphatidylinositol 3-kinases ATM (ataxia telangiectasia mutated), ATR (Ataxia telangiectasia and Rad3 related) and DNA-PK (DNA-dependent protein kinase), can recognize this damage and phosphorylate a multitude of proteins, thus initiating the DNA damage response (DDR). Thereafter, the DDR will initiate the process of either inhibiting cell cycle progression and strengthening DNA repair or directly inducing apoptosis and eliminating the cell, which is regulated by downstream proteins like p53 and breast cancer 1/2 (BRCA1/2) [24].

Telomeres, specialized structures at the end of linear chromosomes, are specifically protected by a nucleoprotein complex known as shelterin. Shelterin sequesters telomeric DNA and prevents it from being recognized as DNA damage, making telomere dysfunction more likely to prompt the aging process and apoptosis [25]. Here, we elucidate the regulation of apoptosis under the pressure of genomic instability from three aspects: nuclear DNA damage, mitochondrial DNA (mtDNA) damage and telomere dysfunction.

### Nuclear DNA damage

The mechanisms of nuclear DNA damage-induced apoptosis in liver aging involve the activation of p38/JNK by direct DNA damage and by deficient DNA repair mechanisms. Methyl methanesulfonate (MMS) is a typical S_N_2 (biomolecular nucleophilic substitution) methylating agent that has been used as a model experimental research chemical to alkylate DNA, causing varying degrees of DNA damage, from single point mutations to double-strand breaks. Suh *et al.* demonstrated that MMS was a strong inducer of brain tumors but a weak hepatocarcinogen, even in the regenerating livers of rats [26]. Interestingly, upon p38 and JNK activation, MMS induced massive apoptosis in the livers instead of in the brains of adult rats, demonstrating the strong correlation between the ability of a tissue to undergo apoptosis and its resistance to carcinogenesis [4]. In this case, MMS signaling induced the phosphorylation of SEK1/mitogen-activated protein kinase kinase 4, which in turn phosphorylated both JNK and p38 to activate downstream targets such as activating transcription factor 2 (ATF2) and c-Jun, finally leading to apoptosis. In subsequent experiments, Suh *et al.* treated young and old rats with a moderate dose of MMS for a short time (one hour), and found that liver cells from the older rats were more resistant to apoptosis in response to genotoxic stress than those of their younger counterparts [27]. The contrast between these two studies might be ascribed to the amount and degree of DNA lesion formation achieved by the methylating agent, depending on the exposure time and dose. In most cases, slight DNA damage is insufficient to trigger apoptosis [24].

DNA repair mechanisms are indispensable for maintaining genomic stability, although they decline with aging [28]. ATM phosphorylates checkpoint kinase-2 (CHK2) in response to the formation of double-strand breaks, while ATR phosphorylates checkpoint kinase-1 (CHK1) in response to stalled DNA replication forks. In turn, CHK2 and CHK1 phosphorylate the transcription factor p53. Then, according to the severity of the damage, p53 regulates the transcription of pro-apoptotic genes like *Fas-R*, *BAX* and p53-upregulated modulator of apoptosis (*PUMA*) or anti-apoptotic genes like damage-specific DNA binding protein 2 (*DDB2)*, xeroderma pigmentosum complementation group C (*XPC)* and flap structure-specific endonuclease 1 (*FEN1)* to decide the fate of the cell [24]. Having a mutation in the *Xpd* gene (R722W), female XPD (TTD) mice exhibit defective nucleotide excision repair and transcription, and display symptoms of premature aging but a reduced incidence of liver cancer. In the livers of these mice, Park *et al.* found that activated caspase-3 and p53 increased with aging, reflecting apoptosis as a compensatory adjustment to limit the increased genotoxic stress in these mutants [29].

### mtDNA damage

mtDNA is a major target of aging-related DNA damage due to the oxidative microenvironment of the mitochondrion, the lack of protective histones in mtDNA and the limited efficiency of mtDNA repair mechanisms compared to those of nuclear DNA [30]. To investigate the interrelationship among mtDNA damage, senescence and apoptosis, Laberge *et al.* applied the synthetic nucleoside analog ganciclovir to create or eliminate several kinds of senescent human cells *in vivo*. The results were consistent with hormesis theory: low concentrations of ganciclovir induced senescence via accumulating nuclear DNA damage, while higher concentrations of ganciclovir killed non-dividing senescent cells via mtDNA damage and caspase-dependent apoptosis [31]. Alongside liver aging, mtDNA damage is multidimensional: the mtDNA content commonly decreases in the livers of aging rats [32]; aging increases mtDNA damage and oxidative stress in the livers of rhesus monkeys [12]; maintaining the stability of the mtDNA content and mitochondrial dynamics contributes to the longevity of rats [33]; and mtDNA mutations can accelerate liver aging in mice by impairing the ROS response and the mitochondrial life cycle [34]. Many studies have demonstrated that mtDNA damage induces apoptosis through the mitochondrial (intrinsic) pathway in multiple human and mouse somatic cell types [35–37].

Despite the prevailing mitochondrial oxidative stress theory of aging, which emphasizes that oxidative stress damages mtDNA, it is also important to highlight that aging-related mtDNA damage can manifest itself in forms other than oxidative damage [38]. Kujoth *et al.* demonstrated that accumulated mtDNA mutations accelerated aging in mice expressing a proofreading-deficient version of mtDNA polymerase gamma. In this case, while increased markers of oxidative stress or defects in cellular proliferation were not detected, mtDNA damage accelerated liver aging and enhanced liver apoptosis, which was accompanied by increased activation of caspase-3 in liver cells [39]. The authors suggested that the “vicious cycle” theory (mtDNA damage provokes respiratory chain dysfunction, leading to enhanced ROS production, which in turn causes further mtDNA mutations) within the mitochondrial oxidative stress theory of aging remains controversial. Regardless of the relationship between oxidative stress and mtDNA damage, it is obvious that mtDNA damage can promote mitochondrial dysfunction, increase the mitochondrial permeability and stimulate the release of pro-apoptotic factors, thus further activating the caspase cascade and inducing intrinsic apoptosis.

### Telomere dysfunction

**Figure 2:**
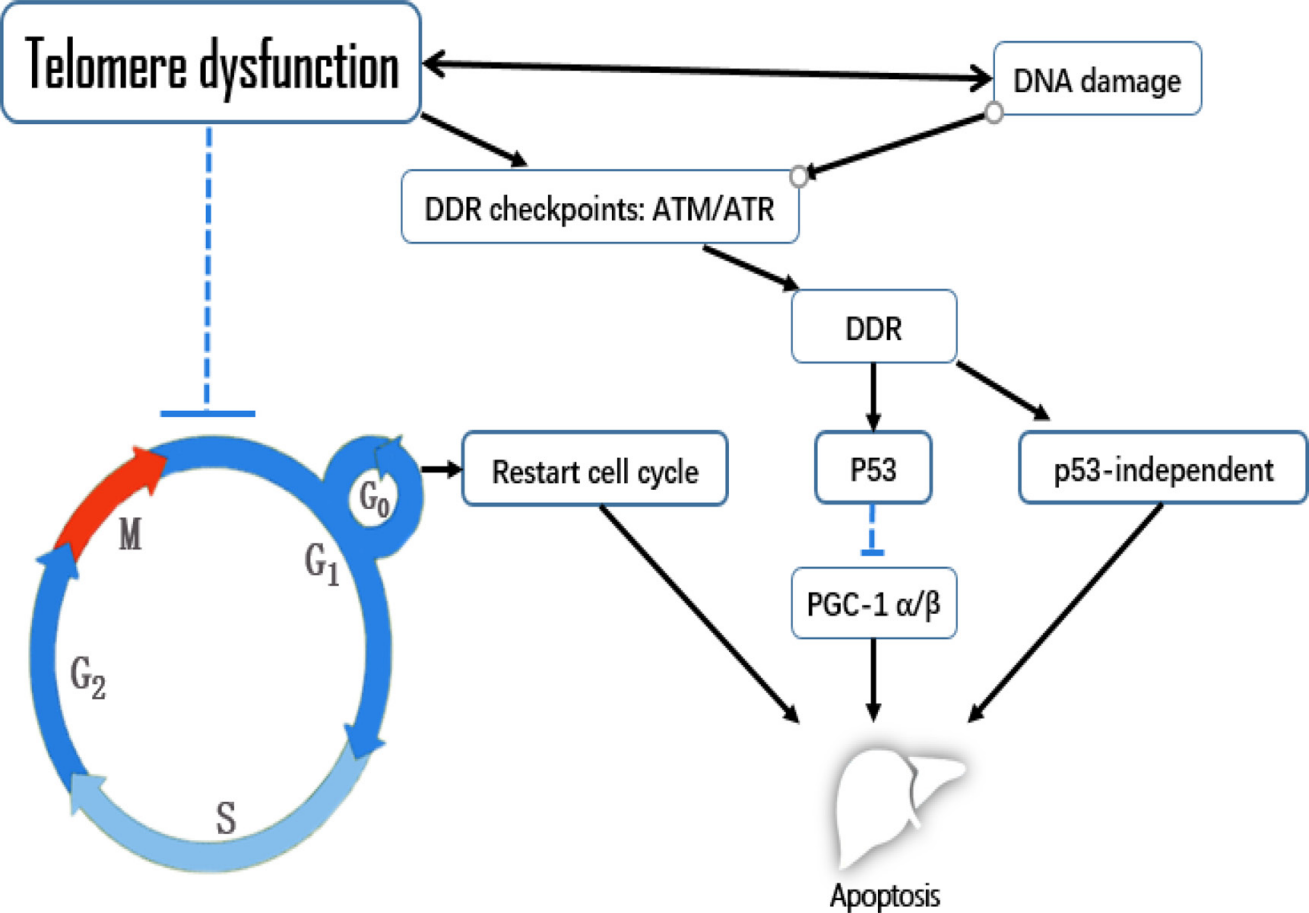
The effects of telomere dysfunction on apoptosis in liver aging

“Telomeres” have long been a major research hotspot in the field of aging. Whether it is the telomere attrition derived from consistent DNA duplication in proliferative cells, or the decline in polymerase activity with aging, telomere dysfunction and cell senescence intersect intimately. In an aging human liver without a history of liver disease, age-related telomere attrition was restricted to Kupffer cells and stellate cells, while cholangiocytes and hepatocytes displayed no age-related telomere shortening [40]. Telomere dysfunction has also been observed in pathologically aging human livers: accelerated telomere attrition and an increased number of senescent cells have been seen in tolerated liver grafts [41], and telomere dysfunction can prompt chronic liver disease and hepatocarcinoma [42]. Especially after the progressive diminishment of shelterin protection, DNA damage in telomeres can be detected easily, and thereafter cells can initiate the DDR to induce apoptosis. Telomere dysfunction-induced apoptosis in liver aging depends on the stage of the cell in the cell-division cycle, and can involve p53-dependent mechanisms or p53-independent mechanisms activated by shelterin dysfunction (Figure 2).

Telomeric repeat-binding factor 2 (TRF2) is a component of the shelterin nucleoprotein complex and a negative regulator of telomere length, the inhibition of which can cause telomere dysfunction. Lechel *et al.* used a dominant-negative version of the protein to inhibit TRF2 in liver cells from 12- to 14-week-old female mice, and found that low inhibition of TRF2 was associated with cellular senescence, whereas high inhibition of TRF2 was associated with apoptosis, as part of a p53-independent mechanism [43]. When Lazzerini *et al.* conditionally deleted *Trf2* in mouse hepatocytes (rather than inhibiting it), p53 was not induced and apoptosis was not detected [44]. The authors postulated that senescence and apoptosis were not detected after *Trf2* deletion because the hepatocytes remained in G_0_ in their experimental setting, while in the TRF2 inhibition experiment, adenoviral infection induced cell cycle entry, which forced hepatocytes into either apoptosis or senescence. The authors from the TRF2 inhibition experiment did not indicate the specific mechanism of this p53-independent apoptosis. However, other research has suggested that, against a background of telomere dysfunction, apoptosis may also be induced by p53-independent mechanisms involving poly (ADP-ribose) polymerase-1 and p73 [45]. A recent study also indicated that p73 is an apoptotic protein that can activate BAX, PUMA and caspase-3 in mouse spleen cells when telomere dysfunction occurs [46].

Telomere dysfunction might also induce apoptosis by causing mitochondrial compromise. For example, peroxisome proliferator-activated receptor gamma coactivator 1 alpha and beta (PGC-1α/β) are master regulators of mitochondrial physiology and metabolism. In liver cells from mice deficient in telomerase RNA component or telomerase reverse transcriptase, Sahin *et al.* demonstrated that telomere dysfunction activated p53, which bound to and repressed the PGC-1α/β promoters, compromising metabolism and mitochondrial function [47]. This study revealed a direct link between telomeres and mitochondrial biology.

Telomere dysfunction mainly impacts the G_1_ phase. Both telomere dysfunction and DNA damage can activate DNA damage response (DDR) checkpoints such as ATM/ATR, which initiate downstream p53-dependent or -independent pathways. p53 can suppress PGC-1α/β, leading to mitochondrial compromise and apoptosis. An unclear p53-independent mechanism of telomere dysfunction in liver aging also promotes apoptosis. In the context of telomere dysfunction, hepatocytes are more likely to initiate apoptosis after re-entering the cell cycle than while remaining in G_0_ phase.

### Lipotoxicity and apoptosis in liver aging

Lipotoxicity is a metabolic syndrome involving excessive accumulation of free fatty acids (FFA), especially saturated fatty acids (SFAs), which can cause liver damage and apoptosis. Epidemiological studies have demonstrated that nonalcoholic fatty liver disease (NAFLD) and non-alcoholic steatohepatitis (NASH) are common among the elderly [48, 49]. Along with aging, insulin resistance reduces hepatic lipid metabolism, causing FFA to accumulate in liver cells. In addition, the decline in antioxidant capacity induces oxidative stress, which produces toxic lipid metabolites that are ubiquitous in NASH [50]. Both of the above cause progressive apoptosis in the liver, which contributes to the pathogenesis of NAFLD. Recent evidence indicates that the severity of NASH is positively associated with apoptosis, and more and more studies support apoptosis as the major mechanism of cell death that drives inflammation and fibrosis in NASH; in fact, apoptosis may be the link between liver injury and fibrosis [51]. This conclusion seems counterintuitive, because apoptosis has always been recognized as a subtle perturbation to the microenvironment. Nevertheless, sterile inflammation driven by apoptosis is important in the pathogenesis of NASH, as excess FFA, oxidative stress and inflammation jointly enhance both extrinsic and intrinsic apoptotic pathways [50]. The mechanisms of lipotoxicity-induced apoptosis in liver aging involve upregulated death receptors, activated p53 and activated NF-κB.

Apoptotic death receptors are highly expressed in lipotoxicity-induced liver injury. In isolated hepatocytes from the steatotic livers of elderly patients, Volkmann *et al.* detected massive extrinsic apoptosis, correlating with increased expression of tumor necrosis factor (TNF)-related apoptosis-inducing ligand (TRAIL) receptors and pro-apoptotic BCL-2 proteins [52]. Malhi *et al.* found that FFA treatment *in vivo* upregulated the cognate TRAIL receptor death receptor 5 in hepatocytes (Huh7 cells, HepG2 cells and primary rat hepatocytes), and this upregulation was JNK-dependent [53]. Another experiment also indicated that death receptors like Fas and TNF-R1 were upregulated in livers from patients with alcoholic steatohepatitis, which aggravated the disease severity, increased hepatocyte apoptosis and correlated with active NF-κB expression [54]. In most cases, NF-κB activation is anti-apoptotic, but it can prompt apoptosis in a cell-type- and stimulus-dependent manner [55]. The authors who conducted this experiment proposed that NF-κB may upregulate death receptors at a transcriptional level in hepatocytes, inducing apoptosis that may override the anti-apoptotic effects of BCL-2 upregulation. Since BCL-2 localizes to the outer membrane of the mitochondrion and inhibits the actions of intrinsic pro-apoptotic proteins like BAX and BCL-2 antagonist/killer 1 (BAK), this experiment suggested that apoptosis in NASH and alcoholic steatohepatitis primarily proceeds through the extrinsic pathway.

Another study concerning NAFLD established that saturated FFA in liver cells can directly induce mitochondrial dysfunction and oxidative stress [56], implying that FFA may also contribute to intrinsic apoptosis. Farrell *et al.* fed mice a methionine- and choline-deficient diet to generate a NASH model. In the liver cells of these mice, p53 was activated, and stimulated mitochondrial apoptosis in three ways: by inhibiting BCL-XL, causing BH3 interacting domain death agonist (BID) to be cleaved to tBID, which migrated to mitochondria to release pro-apoptotic factors; by inducing p21, which inhibited the cyclin D kinase to halt cell proliferation; and by upregulating TRAIL receptor expression, thereby linking the intrinsic and extrinsic apoptosis pathways in NASH [57].

### Endoplasmic reticulum stress and apoptosis in liver aging

The endoplasmic reticulum (ER) is an organelle that is essential for protein folding, lipid biosynthesis and calcium storage. During liver aging, the declining expression and activity of some key ER molecular chaperones and folding enzymes can cause ER stress, in which unfolded and misfolded proteins start to accumulate in cells [58]. As a self-protective mechanism in response to ER stress, the cell may employ the unfolded protein response, which proactively reduces the production of proteins, upregulates molecular chaperones to accelerate protein transportation and folding, and degrades misfolded or useless proteins. However, if ER stress remains unresolved for a long time, cells may apply apoptosis to diminish the detrimental effects of dysfunctional cells on the surrounding microenvironment. The mechanisms of ER stress-induced apoptosis in liver aging involve increased C/EBP homologous protein (CHOP) expression, p53 activation, and reduced sarcoendoplasmic reticulum calcium transport ATPase (SERCA) activity.

CHOP is a pro-apoptotic transcription factor that is expressed at a low level in the normal state but is substantially upregulated under severe ER stress. The expression of the *gadd153* gene (encoding CHOP) was reported to increase with aging in hepatocytes derived from aged rats [59], and CHOP levels were found to be elevated in the livers of five kinds of slow-aging mice [60]. Enkhbold *et al.* investigated the livers of young and aged mice after hepatectomy, and found that pro-apoptotic CHOP levels were greater, anti-apoptotic senescence marker protein 30 (SMP30) levels were lower, and thus the extent of hepatic apoptosis was greater in the older mice than in their younger counterparts [61].

Li *et al.* pointed out that once ER stress activates the unfolded protein response, three ER stress sensor proteins (inositol-requiring enzyme 1 [IRE1], protein kinase R-like ER kinase [PERK] and ATF6) can upregulate the expression of CHOP. If the ER stress is prolonged or overwhelming, overexpression of CHOP can alter the expression of numerous pro- and anti-apoptotic genes, including *DOC* genes (for ‘downstream of CHOP’), *BCL-2* and tribbles-related protein 3 (*TRB3*), thereby activating the apoptotic cascade [62]. Additionally, Cazanave *et al.* demonstrated that in Huh7 cells subjected to FFA-induced ER stress, CHOP physically bound to the activator protein-1 complex protein c-Jun. The resultant heteromeric complex bound to the *PUMA* promoter region, which triggered the mitochondrial pathway of apoptosis [63]. With regard to SMP30, this protein maintains intracellular Ca^2+^ homeostasis by activating Ca^2+^ pump enzymes, and its expression also decreases with liver aging [64]. Ishigami *et al.* found that *SMP30*–/– hepatocytes were highly susceptible to TNF-α- and Fas-induced apoptosis, and suggested that SMP30 may link TNF-α-dependent increases in the intracellular Ca^2+^ concentration with extrinsic apoptosis [65].

SERCA is a pump that transports calcium ions from the cytoplasm into the sarcoendoplasmic reticulum. SERCA expression and activity are reduced in aging and in a range of pathophysiological conditions, including cardiac and skeletal muscle disease [66]. Since increases in ER calcium levels can activate apoptotic effectors such as BCL-2 protein family members [67], Bozaykut *et al.* suggested that reduced SERCA expression causes ER calcium efflux, which results in mitochondrial membrane decomposition and further intrinsic apoptosis [68]. As hepatic SERCA activity was also found to be reduced in an obese murine model [69], aging- and disease-related declines in SERCA activity may contribute to apoptosis during liver aging.

### Dysregulation of nutrient sensing and apoptosis in liver aging

Nutrient sensing is the process in which cells recognize and respond to different environmental nutrient levels, and this process is commonly dysregulated in the aging process. Growth hormone (GH), which is produced by the anterior pituitary, can induce many types of cells (mainly hepatocytes) to secrete insulin-like growth factor (IGF-1), which is similar to insulin either in molecular structure or function, informing cells of the presence of glucose. IGF-1 and insulin signaling are jointly known as the insulin and IGF-1 signaling (IIS) pathway. Another protein related to apoptosis in liver aging is sirtuin 1 (SIRT1), which maintains physiological functions by improving genomic stability, and can be used by aging cells to enhance mitochondrial biogenesis, stress tolerance and fat metabolism. After sensing abnormal nutrient concentrations, IIS and SIRT1 can immediately regulate gene expression and protein modification to help cells adapt to the nutrient stress, thereby avoiding apoptosis. However, the efficiency of IIS and SIRT1 declines with aging [30]. Moreover, they are mediators of the beneficial effects of calorie restriction (CR), which is a universally recognized method of slowing the biological aging process in a range of animals [70]. CR can restore nutrient sensing in aged animals, which might explain why CR suppressed the age-enhanced susceptibility to apoptosis in the livers of male rats [71]. The mechanism of nutrient sensing dysregulation-induced apoptosis in liver aging involves intrinsic apoptosis induced by declines in IIS and SIRT1.

IIS signaling consists of GH, IGF-1 and insulin. After treating aged rats with GH, Tresguerres *et al.* found reduced oxidative stress and apoptosis in their livers [72]. In this case, GH exerted many beneficial effects that reduced oxidative stress: it increased hepatic ATP production, increased the activities of cytosolic antioxidants such as glutathione, reduced mitochondrial nitric oxide levels, and prevented the efflux of mitochondrial cytochrome C that initiates intrinsic apoptosis. As for IGF-1, Puche *et al.* restored circulating IGF-1 levels in aging rats, which normally decline with age. Whereas livers from untreated rats significantly overexpressed the active fragments of caspases-3 and -9, the livers from the aging rats treated with IGF-1 exhibited reversed mitochondrial dysfunction and reduced caspase activation [73]. The authors reported that IGF-1 therapy corrected some parameters of mitochondrial dysfunction, increased ATP production, and thereby reduced free radical production, oxidative damage and apoptosis.

A similar situation was encountered in transgenic alpha MUPA mice, which spontaneously eat less, live longer and have lower serum IGF-1 levels than their wild-type controls [74]. With regard to the simultaneously increased apoptotic capacity exhibited in alpha MUPA livers, Tirosh *et al.* suggested that low levels of IGF-1 impaired mitochondrial function, resulting in mtPTP formation, cytochrome C release and intrinsic apoptosis. Moreover, Kooijman suggested that IGF-1 exerts its anti-apoptotic effects mainly via the phosphatidylinositol 3-kinase-AKT pathway to regulate the expression and activity of BCL-2 family proteins in the intrinsic apoptotic pathway [75].

As for SIRT1, this protein has been found to inhibit apoptosis in human chondrocytes by downregulating mitochondria-related apoptotic signals [76]. Nascimento *et al.* also found that reduced SIRT1 activity in rat livers aggravated NASH and enhanced apoptosis [77]. After activating SIRT1 in conditional *Sirt1* knockout mice with SRT1720, Minor *et al.* found that SRT1720 reduced PGC-1α acetylation in the liver, which increased cell survival and mitochondrial respiration, thereby blocking apoptosis [78].

### Liver stem/progenitor cells (LSPCs) and apoptosis in liver aging

The liver possesses a remarkable regenerative capacity after surgical resection or liver injury, though this capacity declines with aging. LSPCs have the potential to proliferate and differentiate to hepatocytes or cholangiocytes, and are thought to be activated in this process [79]. As research on heterochronic/isochronic parabiosis and cell transplantation has flourished in recent years, there has been increasing interest in the prospect of rejuvenating aging cells by substituting their stem cell environment with that from younger cells. The benefits of LSPCs from younger or healthier individuals have been established in liver aging, and transplanting them into aged livers can even increase apoptosis in adjacent host liver cells. This phenomenon might be attributed to cell competition, in which cells of higher fitness progressively replace less adaptive neighboring cells by inducing apoptosis [80]. The factors influencing the survival and functioning of LSPCs in liver aging can be divided into three categories: niches, systemic factors, and LSPC senescence *per se*. The mechanisms of apoptosis in LSPC-related liver aging involve increased secretion of matrix metalloproteinase-2 (MMP-2), oxidative stress in niches, systemic factors and increased activin A expression.

The specialized microenvironments in which LSPCs reside are called niches, which exchange signals with them to promote cell maintenance and determine cell fate. As niches appear to regulate most of the key functions of stem cells [81], niche cell aging and age-related alterations in the extracellular material in niches can impede stem cell proliferation and differentiation [82]. Oertel *et al.* transplanted fetal LSPCs into normal adult rat livers, which stimulated normal liver reconstitution and increased apoptosis in neighboring host hepatocytes [83]. Since the area surrounding transplanted fetal LSPCs exhibited increased activity of MMP-2, which can be synthesized by hepatic stellate cells, macrophages and hepatocytes [84], it is likely that the increased secretion of MMP-2 (and possibly other MMPs) helped to drive apoptosis in nearby hepatocytes. MMPs are a protease family that can degrade the complex hepatic extracellular matrix and participate in proteolysis that causes parenchymal cells to detach from the complex extracellular matrix [85]. This, in turn, enhances apoptosis, according to evidence from MMP-focused liver studies [85, 86], by altering cell–matrix and cell–cell interactions, and by releasing apoptotic proteins. Additionally, a recent study by Cheng *et al.* revealed that aging hepatic stellate cells activated neutrophils, which produced ROS that infiltrated into liver niches and caused maladaptive changes in liver progenitor cells in old mice, which might have led to stem cell senescence and apoptosis [87].

In addition to altering the local niche environment, aging also alters systemic factors that can profoundly impact LSPCs. Conboy *et al.* restored aged liver progenitor cells by establishing parabiotic pairings (a shared circulatory system) between young and old mice, suggesting that there are systemic factors unique to young mice that can enhance progenitor cell proliferation [88]. Though these factors have not yet been clearly identified, systemic factors could influence local LSPC apoptosis in many ways [89, 90].

The senescence of LSPCs *per se* is coupled with increased apoptosis. Menthena *et al.* found less proliferation and more apoptosis in LSPCs from older rats than from younger rats. This phenomenon in older rats was attributed to increased expression of activin A, a potent growth suppressor that can strongly downregulate anti-apoptotic genes in hepatocytes [91].

### Conclusion and prospects

On the whole, the current literature indicates that apoptosis (whether intrinsic, extrinsic, or other non-classical apoptosis) increases in both normal and pathological liver aging. In this process, internal influential factors like oxidative stress, genomic instability, lipotoxicity, endoplasmic reticulum stress and nutrient sensing dysregulation have been characterized extensively in gerontologic studies. Regarding the cross-talk among these internal factors, it is difficult to place particular weight on any one mechanism of apoptosis in liver aging. What specifically deserves to be mentioned is “hormesis,” which appears in a broad range of stress conditions, and is the phenomenon in which low doses of toxins and other stressors can activate adaptive stress responses that enhance cellular resistance and maintenance, whereas high dose of these agents exceed the processing capacity of cells and result in apoptosis or necrosis [92]. This theory seems to explain liver aging apoptosis in conditions such as oxidative stress and genomic instability. Hence, we compare apoptosis to an immune response: even if apoptosis is a protective mechanism in response to various types of aging-related damage, too much or too little apoptosis is detrimental. For the interest of liver as a whole, a delicate balance of apoptosis should be maintained to achieve the maximum aging delay or the minimum impact of aging on the body.

While the internal factors influencing liver aging have been well-documented, external influential factors such as systemic factors and cell niches still require further investigation. Based on the current evidence, though, it is clear that the local/systemic environment of a young animal can restore the functioning of aged LSPCs in many ways, and that apoptosis is liable to occur in aged liver cells due to cell competition. Regarding more specific details, many questions remain unresolved. First, it is unclear whether the “vicious cycle” between mtDNA damage and oxidative stress indeed exists in liver aging. The mitochondrial theory of aging is partially based upon the idea of a vicious cycle, in which mtDNA damage induced by ROS incites respiratory chain dysfunction and subsequently increases ROS production; however, studies of aging in the liver and other organs do not support this point [38, 93]. Second, some experiments have already revealed a p53-independent mechanism in which apoptosis is induced by telomere dysfunction during liver aging [43, 46], but it is not known which signaling pathways participate in this process. Third, as CR is a promising method to slow down biological aging, it has been proposed that CR promotes longevity by attenuating stress-induced apoptosis [94]; however, the effects of CR on liver aging deserve greater attention. Fourth, since apoptosis has been demonstrated to increase in aged liver tissue restored by young niches, it is worth investigating whether systemic factors provided by heterochronic parabiosis could similarly induce apoptosis.

Simply prolonging the lifespan of an organism would be meaningless if the organism were incapable of maintaining normal physiological functions. However, given the prevalence of liver disease among the aging, and the interrelationship among apoptosis, hepatocarcinoma and liver cirrhosis, it is clinically significant to understand the mechanisms underlying apoptosis in liver aging.
